# Proceedings of the Minnesota Medical Convention, Held at St. Paul

**Published:** 1853-09

**Authors:** 


*Proceedings of the Minnesota Medical Convention, held at St. Paul, July* 23, 1853.

PURSUANT to a previous notice through the newspapers and otherwise, a convention of Physicians in Minnesota, met at the Court House in St. Paul, on July 23, 1853.

On motion of Dr. Ames, of Minneapolis, for a temporary organization, Dr. Thos. Potts, of St. Paul, was called to the chair, and Dr. Chas. L. Anderson, of St. Anthony Falls, was appointed Secretary.

On motion of Dr Ames, it was

*Resolved*, That we, Physicians, of Minnesota, deem it expedient to proceed to the organization of a Medical Society.

On motion, it was also

*Resolved*, That the Convention now resolve itself into a Committee of the whole, to produce a plan for the organization of a Medical Society in Minnesota.

---

PROCEEDINGS IN COMMITTEE OF THE WHOLE—Dr. John H. Murphy in the Chair.—Dr. Ames produced a form for the Constitution and By-Laws, which after duly considering and amending was reported, along with the National Medical Code of Ethics, to the Convention, and the Committee adjourned to meet in Convention at 2 o'clock P.M.

---

AFTERNOON SESSION.

Convention met, and on motion, the report of the Committee was unanimously adopted.

On motion of Dr. Mann of St. Paul, it was

*Resolved*, That medical gentlemen present, of recent residence, be invited to take seats and participate in the deliberations of this Convention.

Dr. Ames then moved that the Convention now resolve itself into the Minnesota Medical Society, which was passed.

On motion, a committee of three was appointed to nominate suitable officers and committees for the society, to serve according to the Constitution and By-Laws for the ensuing year:

*President*—Dr. Thos. Potts, of St. Paul.

*Vice-Presidents*—Dr. John H. Murphy of St. Anthony's Falls, and Dr. E. A. Ames, of Minneapolis.

*Corresponding Secretary*—Dr. T. T. Mann, of St. Paul.

*Recording Secretary*—Dr. Chas. L. Anderson, of St. Anthony's Falls.

*Treasurer*—Dr. J. D. Goodrich, of St. Paul.

*Censors*—Drs. Wm. W. Finch, J. H. Day, and John J. Dewey, of St. Paul.

The following is the report on Committees, which was unanimously adopted:

*Committee of Arrangements*—Drs. Murphy, Anderson and Johnson, of St. Anthony's Falls.

*Committee on Practice of Medicine*—Drs. Brisbine, of St. Paul, Carli, of Stillwater, and Anderson of St. Anthony Falls.

*Committee on Surgery*—Drs. McDougal, of Fort Snelling, J. H. Day, of St. Paul, and Ames, of Minneapolis.

*Committee on Obsterics*—Drs. Potts, of St. Paul, Murphy, of St. Anthony Falls, and Mann, of St. Paul.

*Committee on Drugs and Medicines*—Drs. Goodrich, Willey, and Dewey, of St Paul.

*Committee on Publication*, (*ex officio*)—Drs. Mann, of St. Paul, Anderson, of St. Anthony Falls, and Goodrich of St. Paul.

On motion, the Society then proceeded to ballot for a delegate to the next meeting of the American Medical Association; which resulted in the election of Dr. John H. Murphy, of St. Anthony Falls.

Dr. Ames offered the following resolution:

*Resolved*, That this Society highly approve of the efforts made by the American Medical Association to suppress the sale of sophisticated drugs and medicines. Unanimously adopted.

Dr. Anderson offered the following resolution, which was unanimously adopted:

*Resolved*, That the next annual meeting of this Society be held at St. Anthony Falls.

On motion of Dr. Ames it was

*Resolved*, That the Corresponding Secretary be instructed to inform the American Medical Association of our willingness to co-operate with them.

The following physicians were present and assisted in the organization of the Society:

*Permanent Members.*

Drs. Thos. R. Potts, of St. Paul; Jas. D. Goodrich, do; W. W. Finch, do; Jno. J. Dewey, do; T. T. Mann, do; Jno. H. Murphy. St. Anthony Falls; Chas. L. Anderson, do; A. E. Johnson, St. Anthony City; Alfred E. Ames, Hennepin Co.

*Present by Request.*

Drs. A. W. Daniels; O. P. Marsh.

The following physicians were not present, but had expressed a desire to co-operate with the Society; and for that reason are unanimously elected members:

Drs. J. H. Day, St. Paul; Brisbine, do; Willey, do; McDougal, Fort Snelling; Carli, Stillwater.

After many expressions of hope for the success and usefulness of the Society, and of its members, it adjourned to meet again on the first Wednesday in June, 1854, at St. Anthony Falls.

T. R. POTTS, President.

C. L. ANDERSON,<br>T. T. MANN, } Secretaries.

